# Metagenomic analysis reveals resistome characteristics and high-risk resistance genes in the pig nasal cavities, feces, and farm dust

**DOI:** 10.1186/s42523-026-00589-y

**Published:** 2026-06-12

**Authors:** Yangfan Yu, Hai Wu, Honghu Ji, Yuhan Hu, Yihuan Fang, Yuntian Lin, Yong Zhang, Yunyan Zhou

**Affiliations:** 1 College of Pharmaceutical Science & Collaborative Innovation Center of Yangtze River Delta Region Green Pharmaceuticals, Zhejiang University of Technology, Hangzhou, 310014 China; 2 Jinhua Academy of Agricultural Sciences, Jinhua, 321017 China

**Keywords:** Antibiotic resistance genes, Mobile genetic elements, Pig, Metagenome, Risk assessment

## Abstract

**Background:**

Antimicrobial resistance (AMR) poses a threat to global public health. Swine farms are critical AMR reservoirs. Comprehensive resistome profiling and risk assessment across pig-associated niches remain limited. Metagenomic analysis of antibiotic resistance genes (ARGs) in pig nasal cavities, feces, and farm dust was performed.

**Results:**

Nasal and dust samples exhibited significantly increased ARG diversity and abundance compared with feces. We identified 78 potentially hazardous ARGs and proposed an improved risk classification framework integrating host promiscuity, mobility, and human health risks. These ARGs were classified into four risk levels: 25 Level I (current high risk), 25 Level II (potential future threats), 18 Level III (host-promiscuous but nonmobile), and 10 Level IV (host-specific). High-risk ARGs mainly confer aminoglycoside, macrolide–lincosamide–streptogramin (MLS), and tetracycline resistance. Metagenome-assembled genome (MAG) analysis revealed that bacterial taxa enriched in ARGs were predominant in nasal and dust samples. Moreover, these environments presented higher mobile genetic element (MGE) abundance and similar ARG–MGE co-occurrence patterns. Notably, 74.12% of the mobile ARGs were predicted to be plasmid-borne, and these ARGs tended to be assigned higher health risk levels than chromosomal ARGs.

**Conclusions:**

These findings provide a practical framework for ARG risk assessment and highlight the nasal cavity and dust as underappreciated but important AMR reservoirs in pig farms.

**Supplementary Information:**

The online version contains supplementary material available at 10.1186/s42523-026-00589-y.

## Background

The expanded use of antibiotics has been a critical driver in the thriving development of the pig industry, with animal husbandry contributing to more than 50% of worldwide antibiotic consumption [1]. However, the unreasonable use of antibiotics has concurrently contributed to severe antimicrobial resistance (AMR) issues. The administration of antibiotics in feed—even briefly and at low levels—significantly increases both the number and the diversity of resistance genes [2]. AMR can aggravate gut microbiota dysbiosis, potentially compromising pig health and productivity [3]. More critically, widespread antibiotic exposure accelerates the dissemination of AMR through the selective enrichment of antibiotic-resistant bacteria (ARB) and the mobilization of antibiotic resistance genes (ARGs) within microbial communities [4]. Mobile genetic elements (MGEs), such as plasmids, integrons, integrative and conjugative elements (ICEs), and transposases, play a critical role in the horizontal gene transfer of ARGs, especially under antibiotic selection pressure. MGEs also facilitate the spread of ARGs across different media, such as sludge from antibiotic production wastewater, soil, sediment, and even microplastics [1, 5–8]. Studies have demonstrated that ARB and ARGs enriched in livestock can be transferred to the environment and humans through multiple dissemination pathways. For example, Chen et al. reported that manure fertilization introduces substantial amounts of veterinary antibiotics and ARGs into soil [9], whereas He et al. reported wastewater sludge from livestock operations as a key medium for the dissemination of these contaminants into aquatic systems [10]. These contaminants may alter microbial communities in the environment and increase the risk of human-associated bacteria and human pathogens acquiring resistance through MGEs.

Microbial communities associated with pigs serve as significant reservoirs of ARGs. Current research on the pig resistome focuses primarily on the gut microbiome. Antibiotics are extensively used for respiratory infections in pig production. Recent studies have further characterized resistome profiles in the pig lower respiratory tract [11]. The nasal cavity is also regarded as a key site for host‒pathogen interaction and respiratory disease [12]. However, investigations of the nasal resistome remain relatively limited. Abdullahi et al. analysed nasotracheal enterococcal carriage, the resistome, and zoonotic transmission in pigs and other animals, but failed to comprehensively characterize the nasal resistome using metagenomic approaches [13, 14]. The farm environment (e.g., air, dust, and soil) serves as a key vector for the dissemination of pig-derived ARGs to the wider environment and humans [15–17]. Among these, dust integrates ARGs originating from feces, feed, and other sources, facilitating widespread airborne exposure and linking animal and environmental resistomes. Thus, exposure to farm dust may pose a concern for both animals and humans, including farmers and nearby residents [15]. Some studies have compared the types of ARGs in human, pig and various environmental samples [15, 18]. However, these samples were obtained from different breeding environments or even public data from different studies. Owing to variations in antibiotic usage patterns, swine breeds, feeding conditions, farm scales, and geographical factors across countries and regions, identifying the definitive drivers shaping resistome composition is challenging. This limitation consequently obstructs investigations into direct ARG transmission pathways from pig to environmental or human microbiomes. Therefore, it is necessary to study the spread of ARGs between pigs and the pig-associated environment within a unified ecosystem.

Although ARGs are ubiquitous in various environments, not all genes pose an equal threat [19]. Quantifying AMR issues in different environments and distinguishing between human health risks (such as pathogens acquiring ARGs) and ecological risks (the potential for ARGs to spread among microbiota) remain challenging [20]. Many approaches have been proposed to assess and rank the risks of individual ARGs in various environments. For instance, Zhang et al. developed a framework for assessing the risk levels of ARGs, categorizing them into four risk levels on the basis of the enrichment of ARGs in human-associated environments, gene mobility, and host pathogenicity [21]. Another study performed a large-scale analysis of 4,572 samples from six different habitats and quantitatively assessed the health risks of 2,561 ARGs using four criteria: human accessibility, mobility, pathogenicity, and clinical availability [22]. Similar assessment methods based on pathogenicity and mobility have also been proposed [23, 24]. In addition, Rumi, M. A. et al. developed MetaCompare 2.0, a macrorisk assessment tool that assesses overall resistome risk across samples or environments on the basis of dual-risk assessments of human resistome risk and ecological resistome risk rather than individual ARG-level assessments [20]. Existing assessment frameworks are primarily based on metagenomic samples from various environments, particularly those associated with humans. ARG risk profiles vary across habitats because of differences in microbial composition, antibiotic usage, and human exposure levels. Pigs are a major source of meat consumption for humans and a critical reservoir for ARG emergence and dissemination. However, no systematic risk evaluation has been conducted focusing exclusively on the pig-associated resistome.

To elucidate the diversity and risks of ARGs across pig production environments, we compared the resistomes from three key niches: nasal cavities, feces, and farm dust. Using metagenomic analysis, we characterized the ARG profiles, bacterial hosts, and associated MGEs. We further proposed an improved risk classification framework that incorporates host promiscuity, mobility, and human health risks to identify and prioritize high-risk ARGs from pig-related sources. We hypothesize that nonfecal niches (the nasal cavity and dust) serve as critical reservoirs of mobile ARGs and that our multidimensional framework can improve risk ranking and guide effective AMR mitigation strategies in livestock farming.

## Methods

### Sample collection and DNA extraction

This study utilized two independent datasets: a discovery dataset and a validation dataset. The discovery dataset included 36 samples (nasal and fecal samples from 15 pigs, and 6 farm dust samples), while the validation dataset included 28 samples (including nasal and fecal samples from 10 pigs, and 8 farm dust samples). Samples were collected from two commercial pig farms located in Hangzhou and Taizhou, Zhejiang Province, China. The sampling protocol was approved by the Animal Welfare and Ethics Committee of Jinhua Academy of Agricultural Sciences (No. JHNKY2025-001) due to the involvement of animal handling for nasal swab and rectal feces collection. All experimental pigs were healthy, similar age, and had not been administered any antibiotics for three months prior to sampling. For each pig, nasal swabs were taken from both nares, and fecal samples were collected from the rectum. Dust samples were individually collected from six distinct locations within the pig housing environment, including floors, walls, railings, troughs, fan inlets, and the odor removal room. At each site, visible dust accumulation was wiped using six sterile cotton swabs per sample. Each swab was passed over the surface at least ten times to ensure adequate material for subsequent DNA extraction. Each location corresponded to one separate dust sample, and these samples were processed separately to preserve their individual environmental characteristics.

All samples were immediately stored at − 80 °C until further processing. DNA of fecal samples was extracted using the Mag-Bind Soil DNA Kit (Omega Bio-tek, Norcross, GA, USA) according to the manufacturer’s instructions. DNA from dust samples and nasal samples was extracted using FastDNA™ Spin Kit for Soil (MP Biomedicals, Southern California, USA) following the manufacturer’s instructions. Extracted DNA concentration and purity were assessed with TBS-380 and NanoDrop2000, respectively. The quality of the extracted DNA was checked on a 1% agarose gel electrophoresis.

### Library construction and metagenomic sequencing

DNA samples that passed quality control were used for library construction. The extracted DNA was sheared to an average fragment size of approximately 350 bp using a Covaris M220 ultrasonicator (Gene Company Limited, China). Paired-end libraries were constructed using NEXTFLEX^®^ Rapid DNA-Seq (Bioo Scientific, Austin, TX, USA) and then sequenced on Illumina NovaSeq (Illumina Inc., San Diego, CA, USA) using NovaSeq 6000 S4 Reagent Kit v1.5 (300 cycles) at Majorbio Bio-Pharm Technology Co., Ltd. (Shanghai, China).

### Metagenomic analysis

From the discovery dataset, an average of 15.4 Gb of raw metagenomic sequencing data was generated per sample. Raw sequencing data were processed using Trimmomatic (v0.33) [25] to trim adapters and remove low-quality reads. Then, BWA (v0.7.12) [26] was employed to align the human (GRCh38.p14) and pig (Sscrofa11.1) reference genomes downloaded from NCBI, and matched reads were removed. The filtered reads from each sample were assembled individually into contigs using Megahit (v1.1.1) [27] with a minimum contig length of 500 bp. A total of 9,576,560 contigs were obtained from 36 samples. Assembled contigs were used to predict open reading frames (ORFs) using MetaProdigal (v2.6.3) [28] with default parameters. The non-redundant gene catalog was constructed using CD-HIT (v4.5.7) [29] with an identity and query coverage threshold of 95% and 90%, respectively. Gene abundance for each sample was quantified by aligning clean reads against the gene catalog using BWA (v0.7.12), and the outputs were converted into BAM format with Samtools (v0.1.19) [30]. Gene abundance was normalized as TPM by the formula:

$$\:TPM=\frac{{N}_{i}/{L}_{i}}{{\sum\:}_{j}{(N}_{j}/{L}_{j})}\times\:{10}^{6}$$

where *N*_*i*_ is the number of reads mapped to the i gene, *L*_*i*_ is the length of the i gene, the index j represents all genes listed in the catalog, while i serves as an identifier for a specific gene. The same processing was applied to the validation dataset.

Metagenome-assembled genomes (MAGs) were reconstructed from individual samples using MetaBAT2 (v2.11.1) [31] with default parameters. MAG quality was assessed through CheckM (v1.0.2) [32], retaining only bins meeting thresholds of ≥ 70% completeness and < 5% contamination. These quality-filtered MAGs were subsequently dereplicated using dRep (v3.2.2) with a 99% average nucleotide identity cutoff [33]. Gene prediction was performed with MetaProdigal (v2.6.3), followed by taxonomic annotation via GTDB-Tk (v2.4.0) against the Genome Taxonomy Database [34]. Finally, MAG abundance profiles were estimated based on the Quant_bins module from MetaWRAP (v1.1.0) [35].

### Identification of ARGs and MGEs

All predicted ORFs from the samples were screened for ARG-like sequences by alignment against the SARG database (v3.2) [36] using Diamond (v0.9.22.123) [37] with blastp parameters (e-value < 1e-5 and identity > 70%). SARG database classifies ARGs into two hierarchical levels: type and subtype. The type level represents the antibiotic class targeted by the resistance gene (e.g., tetracycline resistance genes, sulfonamide resistance genes), while the subtype level specifies the individual resistance genes (e.g., *tetA*, *sul1*). The same procedure and parameters were applied to identify the ARGs carried by MAGs. The non-redundant nucleotide sequence catalog was aligned with the Mobile Genetic Elements database constructed by Parnanen et al. [38] via BLASTN (v2.2.30+) [39] to identify MGEs, using a cut-off of 10^− 5^ e-value, 70% identity, and 60% coverage.

### Identification of host bacteria of ARGs

Taxonomic classification of ARG-carrying bacteria was determined with Kraken2 (v2.1.2) [40] using contigs that carried ARGs. Results were visualized as Sankey diagrams with the *networkD3* R package [41]. For ARGs identified from MAGs, the taxonomic annotation of the MAGs was directly assigned as the host bacteria of the corresponding ARGs.

### Mobility analysis of ARGs

ARG–MGE colocalization events were identified when both ARGs and MGEs were detected within a 5 kb genomic window on the same contig. The genomic location of ARGs (plasmid or chromosome) was predicted using PlasFlow (v1.1.0) [42] by analysing the contigs carrying ARGs. Based on this, mobile ARGs were defined as those either located within 5 kb of an MGE or carried on plasmids. The co-occurrence network of ARGs and MGEs was visualized by Cytoscape (v3.10.3) [43].

### Construction of a risk assessment framework

To accurately identify high-risk ARGs from pig environments, we proposed an improved risk assessment framework based on two widely recognized risk assessment models. The first model, developed by the research group of Zhang Tong [21] (hereafter referred to as Zhang Tong’s framework), classifies ARGs into four risk ranks (Rank I-IV) on the basis of their potential human health risk. Notably, this risk ranking system is directly integrated into the SARG database (https://smile.hku.hk/SARGs), allowing direct query of the risk level (e.g., Rank I/II) for each ARG entry. The second model, developed by the research group of Qian Haifeng [22] (is hereafter referred to as Qian Haifeng’s framework), quantitatively evaluates ARG risk based on human accessibility, mobility, pathogenicity and clinical availability, classifying ARG risk level into four risk ranks (Q1-Q4).

The initial screening of potentially hazardous ARGs was based on the Rank I/II ARGs in Zhang Tong’s framework. To further capture the dominant ARG signals in pig-associated niches, we additionally included the top twenty most abundant ARG subtypes identified in the nasal cavity, feces, and dust. This combined strategy yielded 78 non-redundant ARG subtypes, which were designated as potentially hazardous resistance determinants. Subsequent risk classification of these candidate ARGs was performed based on three key criteria: (i) Host promiscuity (non-host specificity): It represents the cross-host distribution potential of ARGs. ARG subtypes detected in two or more different bacterial species were considered non–host-specific, following previous studies that used cross-host occurrence as an indicator of broader ecological distribution and potential dissemination [44, 45]. (ii) Mobility: It represents the potential for horizontal gene transfer of ARGs, that is, whether the ARG subtype is located within 5 kb of an MGE or carried on plasmids. The 5 kb distance threshold has been widely used to infer potential ARG–MGE linkage, as physical proximity increases the likelihood of co-transfer events [22, 45]. Plasmid carriage was also included as a key indicator of mobility, as multiple studies have demonstrated that plasmid-borne ARGs possess higher dissemination potential [24, 46]. (iii) Human health risks: This criterion evaluates ARG threats to humans based on human accessibility and pathogenicity potential derived from Qian Haifeng’s framework and complemented by our dataset. ARGs with human accessibility exceeding the mean value across all ARGs in that framework, and with pathogenicity potential defined as a pathogenicity score > 0 in Qian Haifeng’s framework or detection in pathogenic bacterial hosts within our dataset, were considered to have elevated human health relevance [22]. Based on the integration of these three criteria, we classified the candidate potentially hazardous ARGs into four risk levels (I-IV), with Level I representing the highest-risk category (Fig. 2a and Table S8).

### Analysis of the abundance and prevalence of ARGs in large-scale pig metagenomic samples

We further assessed the 78 candidate ARGs in 610 fecal and 745 respiratory tract metagenomic samples. Fecal samples were from two public datasets, including 185 samples from 181 pig herds across 9 European countries [47] and 425 samples from 8 different pig farms in China [48]. These two datasets were combined as the fecal dataset. The 745 respiratory tract samples were from 675 experimental pigs across five wild and domesticated pig populations [11]. To address potential differences in ARG identification and quantification pipelines across studies, we normalized the ARG abundances in the original study to relative abundance for further analysis. Furthermore, to minimize the influence of methodological discrepancies—such as the use of different reference databases—we deliberately avoided direct quantitative comparisons of abundance values between datasets. Instead, our analysis focused on the distribution frequency of potentially high-risk ARGs in each public dataset and assessed if their abundance was higher than the average level of all ARGs. The prevalence of ARG subtypes represents the detection frequency of a specific ARG in all samples of a given dataset (fecal or lower respiratory tract).

### Statistical analysis and visualization

Statistical analysis and visualization employ multiple methods, including R (v4.4.2), ImageGP 2 [49], and OriginPro 2021. The α-diversity index of ARGs compositions in different sample types was calculated using the *vegan* R package [50]. The Venn diagram, principal coordinate analysis (PCoA), and heatmaps of the composition of ARG types and subtypes among the nasal cavity, feces, and dust were visualized by ImageGP 2. Boxplots were generated via the *ggpubr* R package [51]. Comparisons between the nasal cavity and feces were performed using the Wilcoxon signed-rank test because these samples were collected from the same pigs. Comparisons between dust and either nasal cavity or fecal samples were performed using the Wilcoxon rank-sum test (Mann-Whitney U test), as dust samples were treated as independent. The genomic location of different ARG types and their distribution in four rank levels were shown via barplots and Sankey diagrams by OriginPro 2021. The *pheatmap* R package [52] was employed to generate the heatmap of ARG distribution of six MAGs harboring more than five ARGs. The Cytoscape software (v3.10.3) [43] was used to visualize the network relationship between ARGs and MGEs, as well as between ARGs and MAGs.

## Results

### Comparison of ARG composition across nasal, fecal, and dust samples

**Fig. 1:**
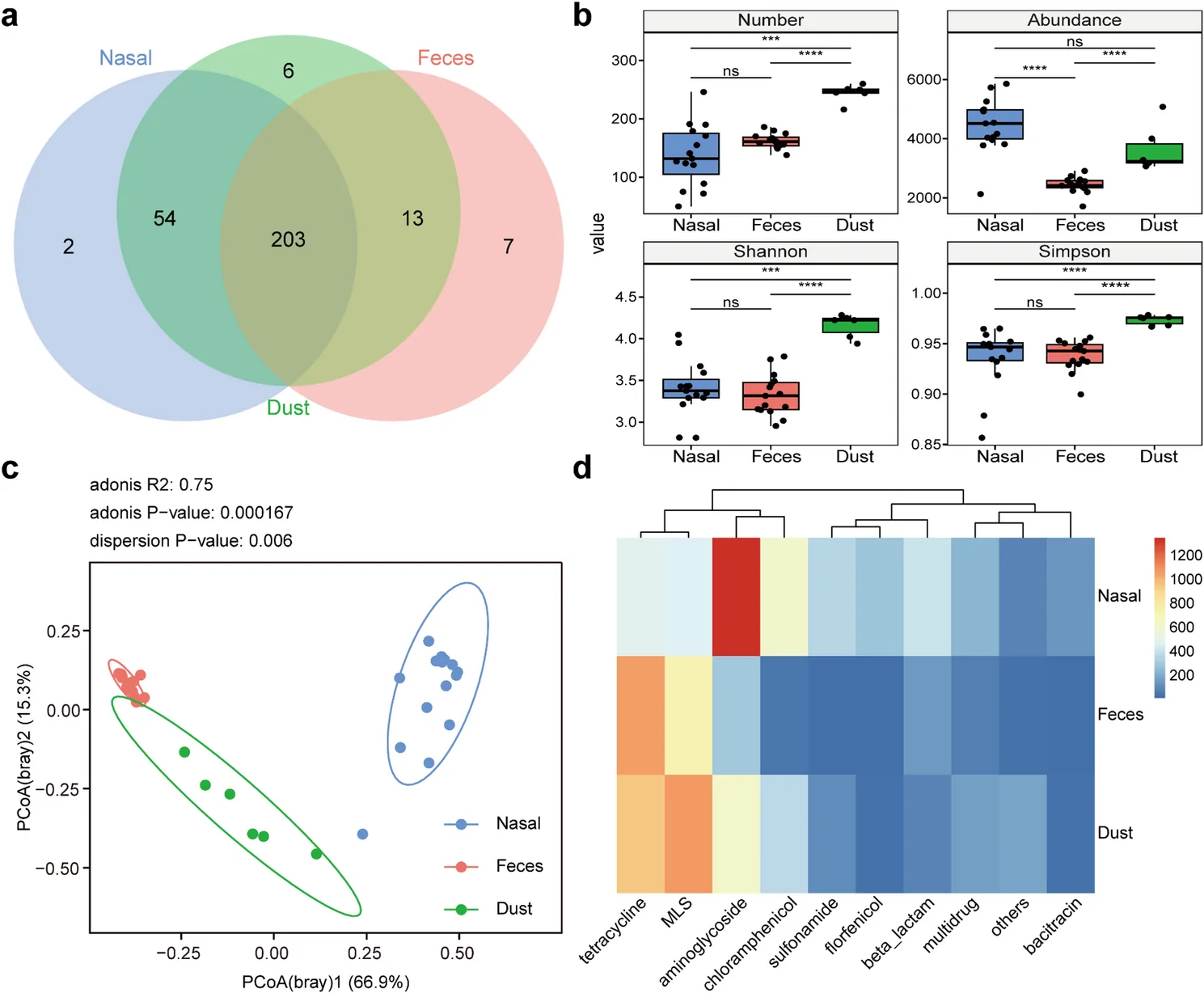
Comparison of ARGs compositions across nasal, fecal, and dust samples. (**a**) Shared and specific ARG subtypes (*n* = 285) among nasal, dust, and feces samples. (**b**) Comparison of the richness (number), abundance, and α-diversity of ARG subtypes between different niches. *p* values were calculated using the Wilcoxon signed-rank test for comparisons between the nasal cavity and feces, and the Wilcoxon rank-sum test for comparisons involving dust. Asterisk indicates significance (*: *p* < 0.05; **: *p* < 0.01; ***: *p* < 0.001; ****: *p* < 0.0001). ns, no significant (*p* ≥ 0.05). (**c**) Principal Co-ordinates Analysis (PCoA) based on Bray-Curtis distance. Each point represents a sample, and the circle within indicates the 95% confidence interval. The P-value (Adonis, *p* < 0.05) indicates significant distinct in the composition of ARGs across different niches. (**d**) The abundance of ARG types among nasal, dust, and feces samples. The figure displays only the top 10 most abundant ARG types, with all others grouped into a single “others” category

A total of 36 samples—comprising 15 nasal swabs, 15 fecal samples, and 6 dust samples—were collected from a commercial pig farm. Metagenomic analysis revealed 4354 predicted ORFs as ARG-like sequences (carried by 3754 contigs), representing 285 ARG subtypes (classified into 23 types) (Table S1). Strikingly, 203 (71.2%) ARG subtypes were shared among the nasal, fecal, and dust samples, whereas only 5.3% of the ARG subtypes were unique to each sample type (Fig. 1a). This extensive overlap in ARG composition indicates strong similarity among niches and suggests possible ecological connectivity between animal- and environment-associated resistomes in swine production systems.

The α-diversity of the ARG subtypes (measured by the richness, Shannon index, and Simpson index; Fig. 1b) was highest in the dust samples, supporting dust as an ARG reservoir. Despite being collected from six distinct environmental locations within the pig housing facilities, all dust samples exhibited similar ARG compositional profiles (Fig. 1c). Nasal samples presented the highest ARG abundance (with an average of 4446 TPM) despite lower α-diversity, suggesting the selective enrichment of specific resistance determinants (Fig. 1b). Although the α-diversities of the nasal and fecal samples were similar (Fig. 1b), their ARG profiles significantly differed (Adonis, *p* = 1.67 × 10^− 4^; Fig. 1c). Nasal samples were enriched in genes conferring resistance to aminoglycoside and chloramphenicol, whereas fecal samples presented higher abundances of macrolide–lincosamide–streptogramin (MLS) and tetracycline resistance genes (Fig. 1d and Fig. S1). Notably, high-abundance ARG types found in nasal and fecal samples were also consistently detected in dust, suggesting that dust may serve as a potential environmental intermediary linking ARG profiles across pig-associated niches.

### Distribution of potentially hazardous ARGs in different niches

Among the identified ARG types, five categories contained more than 50 ARG subtypes, namely, multidrug (170 subtypes), tetracycline (113 subtypes), MLS (107 subtypes), bacitracin (57 subtypes), and aminoglycoside (51 subtypes). By applying the ARG risk assessment framework established by Zhang et al. (Zhang Tong’s framework) [21], we identified 30 Rank I and 26 Rank II ARG subtypes (Table S1). Among these genes, resistance to aminoglycosides and MLS was most prevalent among the Rank I ARGs, whereas tetracycline resistance genes constituted the largest group among the Rank II ARGs. Although multidrug resistance genes represented the most diverse category (170 subtypes), only three were classified as Rank I or II (Fig. S2a). Notably, high-risk ARGs (Rank I and II, considered ‘current threats’ and ‘future threats’, respectively) were widely shared among nasal, fecal, and dust samples. Specifically, 41 of the 48 Rank I ARGs (85.4%) were detected across all three sample types, with only 3 being unique to the fecal and dust samples. Similarly, 22 of the 33 Rank II ARGs (66.7%) were ubiquitous across all sample types, whereas only two were fecal-specific (Fig. S2b). The high degree of overlap in high-risk ARG profiles suggests possible ecological connectivity of clinically relevant ARGs across niches within swine farms. Strikingly, Rank I ARGs constituted 31.2% and 26.0% of the total ARGs in nasal and dust samples, respectively, indicating that these niches may represent important reservoirs of clinically relevant resistance genes (Fig. S2c). Together, these findings support the value of targeted surveillance of nasal and dust resistomes for monitoring ARG-related risks in pig farms.

High-risk ARGs exhibited niche-specific distribution patterns (Fig. S3). *APH(3)-Ib* and *APH(6)-Id* (conferring resistance to aminoglycoside antibiotics), along with the *floR* gene (conferring resistance to chloramphenicol antibiotics), were significantly abundant in nasal samples. *APH(3)-IIIa*, which confers resistance to aminoglycoside antibiotics, was notably enriched in the fecal samples, whereas *erm*(C), *fexA*, and *erm*(B), which confer resistance to MLS antibiotics, were significantly more abundant in the dust samples (Fig. S3a). This niche partitioning was also observed for Rank II ARGs (Fig. S3b). Nasal samples exhibited elevated levels of the *ROB-1* beta-lactamase gene, whereas fecal samples were enriched in tetracycline resistance genes (*tet*(W), *tet*(40)) and MLS (*erm*(F)). The dust samples showed a predominant enrichment in tetracycline resistance genes (*tet*(W), *tet*(40), *tet*(Z), and *tetA*(P)), along with a relatively high abundance of sulfonamide (*sul1*) and MLS (*erm*(F)) resistance genes.

On the basis of the criterion of ranking within the top 20 in terms of average abundance in at least one of the three sample types (nasal cavity, feces, or dust), we identified 44 ARG subtypes and defined them as high-abundance ARGs. These 44 ARGs accounted for 80.1%, 85.2%, and 61.9% of the total ARG abundance in the nasal, fecal, and dust samples, respectively (Fig. S4), indicating that this criterion captured the dominant ARG signals in pig-associated niches. However, not all high-abundance ARGs are high-risk ARGs and that their risk classifications differ between Zhang Tong’s and Qian Haifeng’s risk assessment frameworks. Notably, *cmx*—the most abundant ARG in nasal and dust samples—was classified as a Rank IV ARG in Zhang Tong’s framework but as Rank II (Q2) in Qian Haifeng’s framework [22]. In the fecal samples, the most abundant ARG, *tet*(Q), along with other high-abundance ARGs, such as *tet*(40), *mel*, and *erm*(F), was assigned to Rank II/III in Zhang Tong’s framework but was classified as Rank I (Q1) in Qian Haifeng’s framework. Similarly, dust-enriched ARGs, including *erm*(C), *fexA*, *tet*(L), and *erm*(B) were classified as Rank I in Zhang Tong’s framework, whereas *tet*(W)—Ranked II in Zhang Tong’s framework—was upgraded to Rank I in Qian Haifeng’s framework (Fig. S3c). These discrepancies underscore the complexity of ARG risk evaluation. Moreover, the overall higher-risk assignments under Qian Haifeng’s framework highlight the influence of assessment criteria on ARG risk stratification.

### Reclassification of potentially hazardous ARGs

**Fig. 2:**
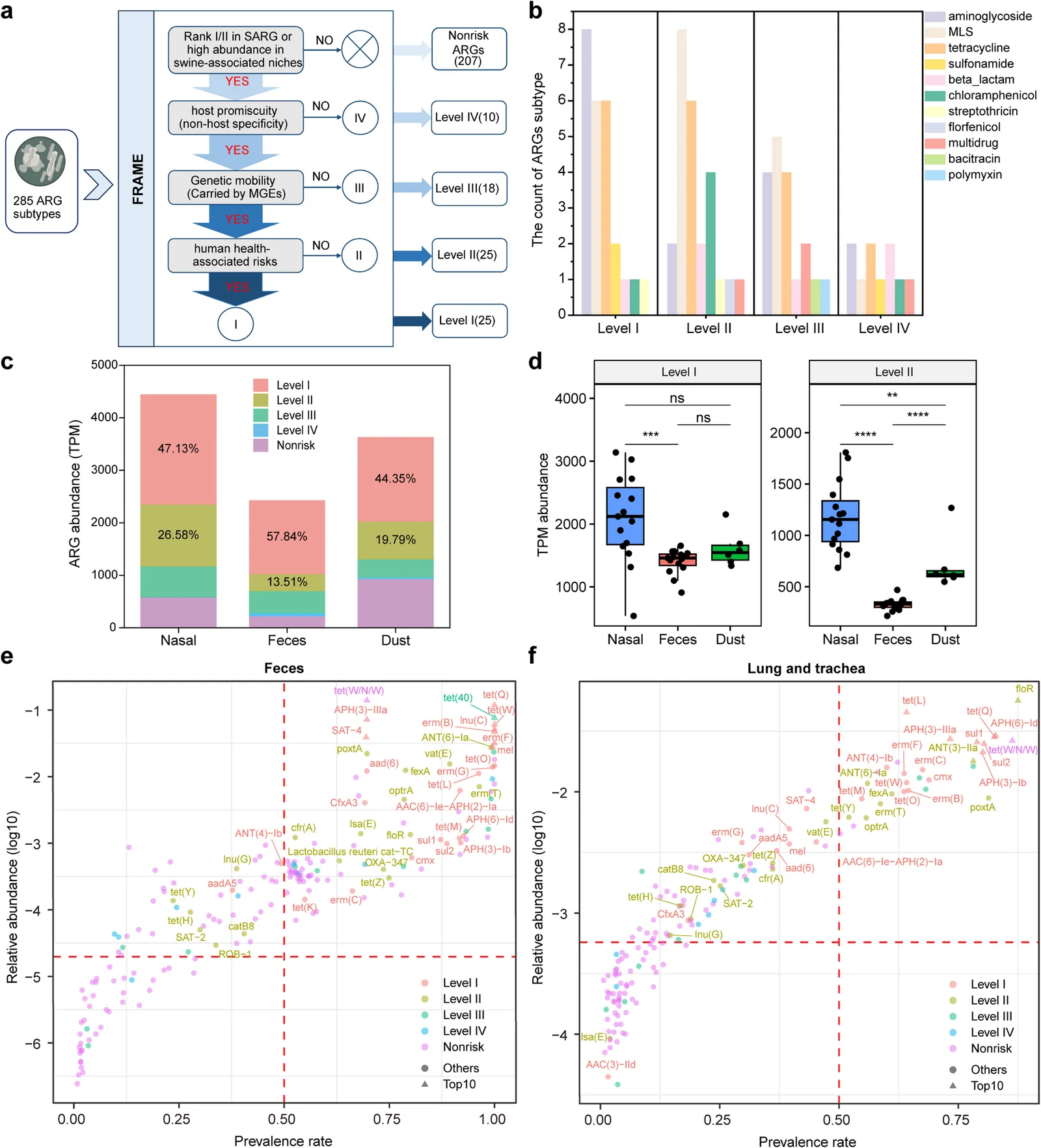
The reclassification of potentially hazardous ARGs. **a.** The framework of the ARG risk assessment. 78 potentially hazardous ARGs were classified into four levels (Level I-IV) based on three criteria: (1) host promiscuity (non-host specificity), (2) mobility, and (3) human health risks (human accessibility and pathogenicity). **b.** The distribution of ARG types within the four risk levels.**c.** The distribution of different ARG risk levels across the three niches under the new classification framework. **d.** The abundance comparison of high-risk ARGs (Level I/II) across three niches. *p* values were calculated using the Wilcoxon signed-rank test for comparisons between the nasal cavity and feces, and the Wilcoxon rank-sum test for comparisons involving dust. Asterisk indicates significance (*: *p* < 0.05; **: *p* < 0.01; ***: *p* < 0.001; ****: *p* < 0.0001). ns, no significant (*p* ≥ 0.05). **e.** The prevalence and abundance of all ARGs under the swine fecal expanded dataset (*n* = 610). **f.** The prevalence and abundance of all ARGs under the respiratory tract (lung and tracheal) expanded dataset (*n* = 745). To reduce data skewness, the relative abundance values were log10-transformed. The triangle represents the top 10 ARG subtypes in terms of abundance. And ARG subtypes with a prevalence greater than 50% and an abundance greater than the average abundance were defined as high prevalence and high abundance (HPHA)

ARG risk classification is framework-dependent. Both the Zhang Tong’s and Qian Haifeng’s frameworks primarily assess human health risks; however, some high-risk ARGs in humans may not originate from swine farms. Therefore, identifying pig-associated high-risk ARGs is critical for developing targeted AMR control strategies in pig production systems. We combined 44 high-abundance ARGs with the Rank I/II ARGs from Zhang Tong’s framework, yielding 78 non-redundant potentially hazardous ARGs. Together, these 78 ARGs accounted for 86.9%, 90.8%, and 74.4% of the total ARG abundance in the nasal, fecal, and dust samples, respectively (Fig. S4), indicating that this candidate set captured the majority of ARG signals across pig-associated niches. We reclassified these 78 potentially hazardous ARGs into four risk levels on the basis of three key criteria: (1) host promiscuity (i.e., non-host specificity), (2) mobility, and (3) human health risks (human accessibility and pathogenicity) (Fig. 2a; see Methods for details). ARGs with host specificity were assigned to Level IV. Those with host promiscuity but lacking mobility were categorized as Level III. Host-promiscuous and mobile ARGs without clear human health risks were assigned to Level II (potential future threats). ARGs that were host-promiscuous, mobile, and posed human health risks were designated as Level I (the highest-risk, current threats). On the basis of this framework, 25 ARGs were classified as Level I, 25 as Level II, 18 as Level III, and 10 as Level IV. MLS and tetracycline resistance genes represented the predominant types across Level I, Level II, and Level III ARGs. Additionally, Level I and Level III contained a greater number of aminoglycoside resistance genes, whereas Level II had more chloramphenicol resistance genes than the other risk levels (Fig. 2b). In addition, we compared the classification results of the 78 potentially hazardous ARGs identified in this study with those derived from Qian Haifeng’s framework and Zhang Tong’s framework. Of the Level I ARGs identified in this study, 21 (84%) overlapped with the Q1 category in Qian Haifeng’s framework (Table S8). However, substantial differences were observed among the three risk assessment frameworks overall (Fig. S5), highlighting the framework-dependent nature of ARG risk classification and the importance of developing evaluation approaches tailored to pig-associated systems.

On the basis of the new ARG risk classification results, we analysed the distribution of ARGs across different risk levels in the nasal cavity, feces, and dust. High-risk ARGs (Level I/II) were highly abundant across all three niches (64.14–73.71%) (Fig. 2c). Moreover, the abundance of both Level I and Level II ARGs was significantly greater in the nasal cavity than in feces, whereas the abundance of Level II ARGs was significantly greater in dust than in feces (Fig. 2d). These findings indicate that high-risk ARGs are widely distributed across swine farm-associated niches and may be well adapted to both host- and environment-associated compartments, particularly highlighting the importance of nasal cavities and dust in the ecology of clinically relevant resistance genes within swine farm environments.

To contextualize our findings within a broader pig microbiome landscape, we analysed the abundance and prevalence of 207 nonrisk ARGs and 78 potentially hazardous ARGs using expanded resistome datasets from 610 swine fecal and 745 respiratory tract (lung and tracheal) metagenomes, notwithstanding the limited sample size in our original study. The results indicated that the majority of nonrisk ARGs (157 out of 207, 75.8%) were present in fewer than 50% of the samples across both the fecal and respiratory tract datasets (Fig. 2e and f). Among these, 69 ARGs were not detected in either dataset. Only three nonrisk ARGs exhibited a detection frequency exceeding 50% in both datasets. In contrast, the 78 potentially hazardous ARGs showed a significantly broader distribution across niches. Specifically, 68 genes were detected in both the pig fecal and respiratory tract datasets, with 56 shared between both niches. Notably, these 56 shared ARGs exhibited a decreasing gradient across risk levels: 92% of Level I (23/25), 89% of Level II (17/19), 71% of Level III (10/14), and 60% of Level IV (6/10) (Fig. 2e and f), suggesting that higher-risk ARGs tend to be more widely distributed across gut and respiratory niches. Overall, the majority of potentially hazardous ARGs were highly prevalent and abundant (HPHA) in both the fecal and respiratory samples. Although several ARGs were relatively low prevalence (< 50%) in respiratory tract samples, their abundance remained notably elevated (above the mean abundance of all the ARGs). These findings indicate that most potentially hazardous ARGs identified by our screening are enriched in swine populations. However, some Level III or even Level IV ARGs also exhibited HPHA profiles (Fig. 2e and f). This pattern suggests that abundance and prevalence do not strictly correlate with risk levels, underscoring the need for multicriteria frameworks in ARG risk assessment.

### Distribution of ARG-harboring bacterial hosts

**Fig. 3:**
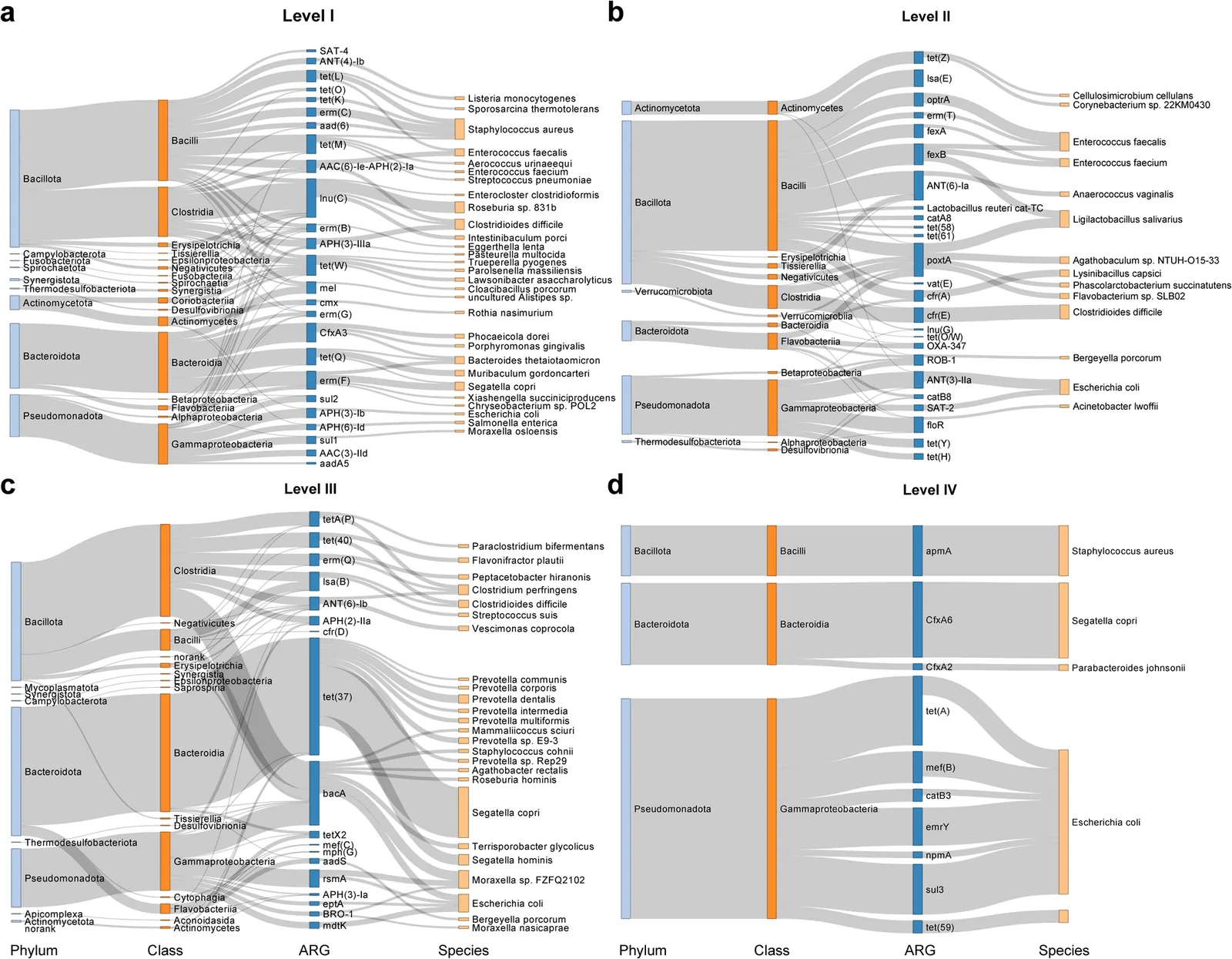
The distribution of host bacteria for ARGs at four risk levels (**a-d**). The lengths of the rectangles indicate the number of ARGs carried by the taxa. Only the bacterial species carrying more than 5 ARGs were presented in the Sankey diagram

The host bacteria of risk ARGs were analysed at different taxonomic levels to understand their distribution and potential for dissemination (Fig. S6). At the phylum level, the primary host bacteria of the ARGs were Bacillota, Pseudomonadota, and Bacteroidota. At the genus level, the dominant hosts included *Escherichia*, *Staphylococcus*, *Segatella*, *Enterococcus*, *Streptococcus*, *Clostridiaceae*, *Clostridium* and *Prevotella*. At the species level, several bacterial species had a high diversity of ARGs across all the samples. For instance, more than 100 ARGs were detected in contigs taxonomically assigned to *Escherichia coli* (563 ARGs), *Segatella copri* (196 ARGs), *Staphylococcus aureus* (195 ARGs), *Clostridioides difficile* (108 ARGs), *Staphylococcus cohnii* (104 ARGs), and *Enterococcus faecalis* (100 ARGs) (Table S2). Notably, these bacterial species harbored diverse ARG types, including Level I or Level II ARGs (Fig. 3), highlighting their potential as vectors for AMR dissemination.

Of the 78 potentially hazardous ARGs, 66 were detected in multiple genera, including 27 distributed across different phyla, whereas only 10 were species-specific and 2 were detected in multiple species within the same genus (Table S8). The host distribution of ARGs varied markedly across the four risk categories (Fig. 3). Level I ARGs—such as *tetM*, *tet*(W), *lnu*(C), *APH(3)-IIIa*, and *erm*(F)—were broadly distributed across diverse bacterial species, spanning multiple classes and even phyla, indicating high host adaptability and dissemination potential (Fig. 3a). Level II and III ARGs also exhibited multiple hosts, although their host ranges were more taxonomically constrained and largely confined to a single bacterial class (Fig. 3b and c). Notably, *poxtA*, *bacA*, and *tet*(37), although not currently classified as human health risks, exhibited broad host ranges across distinct phyla, suggesting the potential for horizontal gene transfer. In contrast, Level IV ARGs were host-specific, were detected exclusively in single bacterial species, and were predominantly carried by Gammaproteobacteria, particularly *Escherichia coli* (Fig. 3d).

### ARG-enriched bacterial taxa based on MAG analysis

**Fig. 4:**
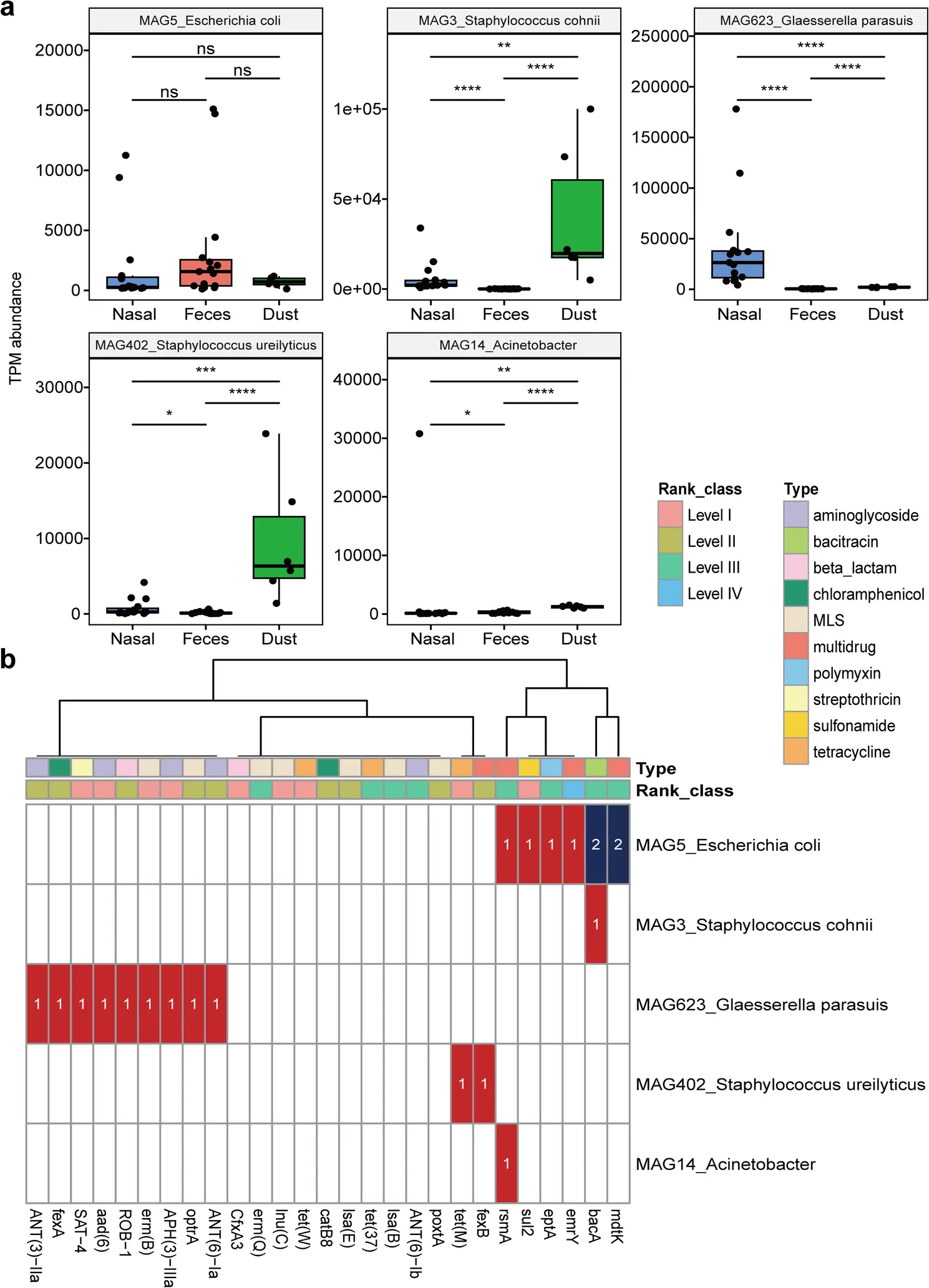
Host bacteria for potentially hazardous ARGs at the metagenome-assembled genome (MAG) level. (**a**) Abundance comparison of MAGs carrying numerous in the nasal cavity, feces and dust. *p* values were calculated using the Wilcoxon signed-rank test for comparisons between the nasal cavity and feces, and the Wilcoxon rank-sum test for comparisons involving dust. *p* < 0.05 was set as the significance threshold. Asterisk indicates significance (*: *p* < 0.05; **: *p* < 0.01; ***: *p* < 0.001; ****: *p* < 0.0001). ns, no significant (*p* ≥ 0.05). Boxplots show the median, 25th, and 75th percentiles. The lower and upper boundaries of whiskers indicate the minima and maxima, respectively. Each dot represents a sample. (**b**) The distribution of the potentially hazardous ARGs within the focused MAGs. Grid values in the heatmap represent the number of each ARG carried by the MAG, and white grids indicate absence

A potential limitation of gene-centric analyses is that the number of ARGs detected in a species may be influenced by its abundance rather than its intrinsic capacity to carry ARGs. To address this issue, we analysed the distribution of ARGs at the MAG level, allowing for a more accurate assessment of ARG-carrying bacterial hosts by focusing on individual genomes rather than overall abundance. We analysed 321 nonredundant MAGs assembled from 36 samples, among which 82 MAGs were identified to harbor ARGs. Five MAGs harbored more than five ARGs, including those from *Escherichia coli* (MAG5: 56 ARGs), *Staphylococcus cohnii* (MAG3: 17 ARGs), *Glaesserella parasuis* (MAG623: 10 ARGs), *Staphylococcus ureilyticus* (MAG402: 8 ARGs), and *Acinetobacter* (MAG14: 6 ARGs) (Table S3). The abundance of the five ARG-rich MAGs varied significantly across sample types (Fig. 4a). *Glaesserella parasuis* (MAG623), a known porcine respiratory pathogen causing Glässer’s disease, was most abundant in the nasal cavity. The abundance of *Escherichia coli* (MAG5) was similar across the nasal cavity, feces, and dust, suggesting its widespread distribution in pig-related environments. The other three MAGs (*S. cohnii*, *S. ureilyticus*, and *Acinetobacter*) were predominantly enriched in dust. Notably, the abundance of *S. cohnii* and *S. ureilyticus* was significantly greater in nasal samples than in fecal samples (Wilcoxon signed-rank test, *p* < 0.05).

Among the 78 potentially hazardous ARGs, 27 were detected and distributed across 54 MAGs (Table S4). Among these genes, *tet*(37), *bacA* and *rsmA* were identified in more than ten MAGs, and *lnu*(C) was identified in four MAGs (Fig. S7). In addition, *bacA* was widely distributed across multiple genera, including *Moraxella*, *Psychrobacter*, and *Agathobacter*, whereas *lnu*(C) was also found in diverse genera. In contrast, *tet*(37) was detected mainly in *Prevotella*, which is consistent with its genus-preferential distribution pattern observed in Fig. 3c. We further analysed the distribution of these 27 ARGs in five ARG-rich MAGs. Among the MAGs analysed, *Glaesserella parasuis* (MAG623) harbored the greatest number of high-risk ARGs (Fig. 4b), including four Level I ARGs—*aad*(6), *SAT-4*, *APH(3)-IIIa*, and *erm*(B)—conferring resistance to aminoglycosides, streptothricin, and macrolides. It also carried five Level II ARGs—*optrA*, *ANT(6)-Ia*, *ROB-1*, *fexA*, and *ANT(3)-IIa*—linked to resistance against oxazolidinones, aminoglycosides, beta-lactams, and streptogramins. In contrast, although *Escherichia coli* (MAG5) carried the greatest number of ARGs (56), only eight were classified as potentially hazardous, with just one Level I ARG (*sul2*) (Fig. 4b).

### Distribution characteristics of MGEs in nasal, fecal, and dust samples

**Fig. 5:**
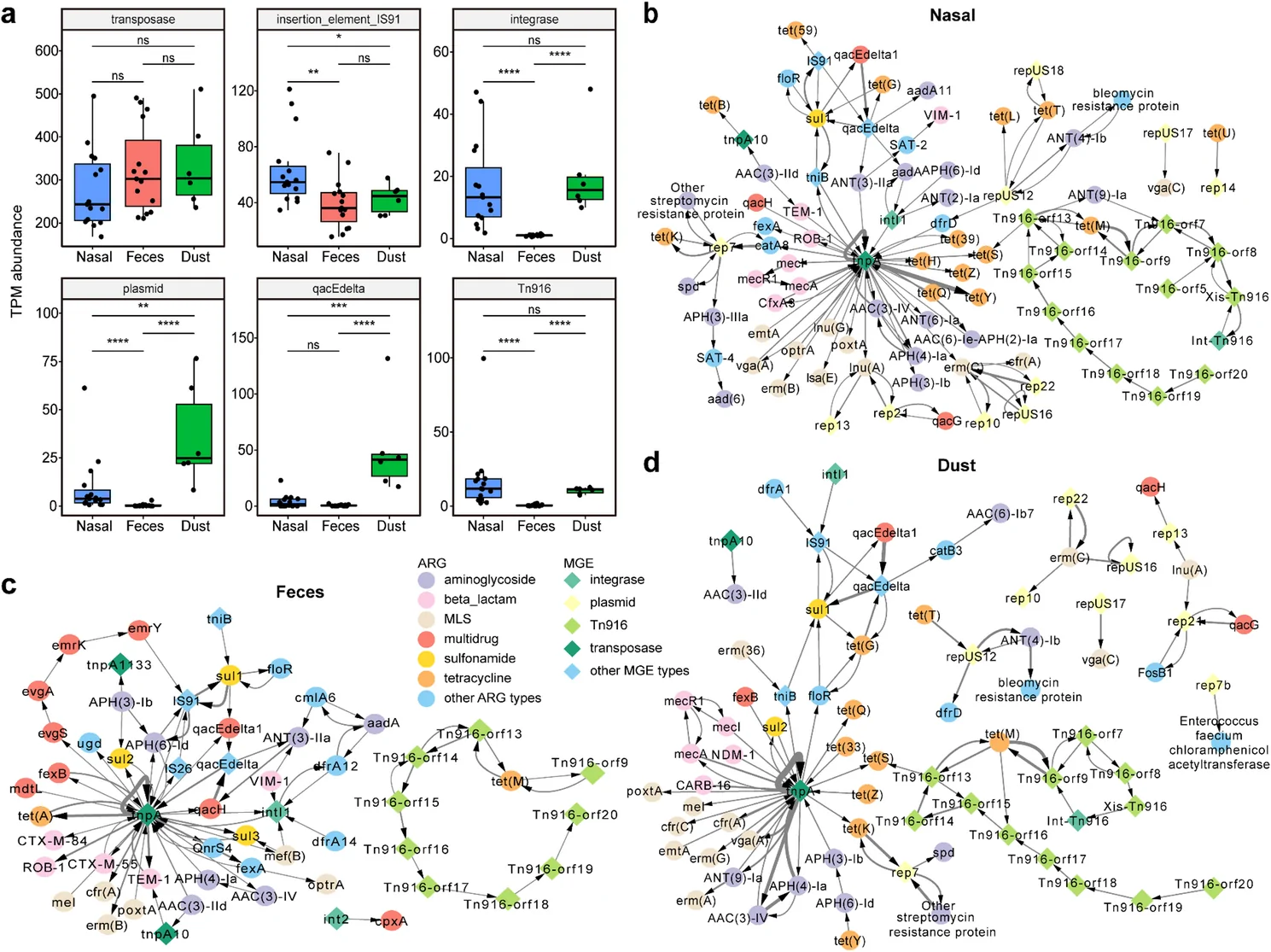
Comparison of MGE types among the nasal cavity, feces and dust and the co-occurrence network of ARG-MGE. **a.** Comparison of MGE types among the nasal cavity, feces and dust. *p* values were calculated using the Wilcoxon signed-rank test for comparisons between the nasal cavity and feces, and the Wilcoxon rank-sum test for comparisons involving dust. *p* < 0.05 was set as the significance threshold. Asterisk indicates significance (*: *p* < 0.05; **: *p* < 0.01; ***: *p* < 0.001; ****: *p* < 0.0001). ns, no significant (*p* ≥ 0.05). **b-d.** The co-occurrence network of ARG-MGE in each niche. The circle represents ARG, the quadrilateral represents MGE, and colors represent different types of ARG or MGE. The direction of the arrow represents the upstream and downstream relationship of the two genes, and the thickness represents the frequency of co-localization of ARG-MGE

MGEs play important roles in the dissemination of ARGs. In the 36 analysed samples, a total of 3316 predicted ORFs were identified as MGE-like sequences, which were distributed across 2750 contigs (Table S5). After duplicates were removed, 551 nonredundant MGEs were classified into 13 major MGE types and 68 subtypes. The dominant MGE types included transposase (75%), insertion element IS91 (12%), integrase (3%), plasmid (3%), qacEdelta (3%), and transposon Tn916 (2%) (Fig. S8). Comparative analysis revealed that nearly all MGE types (except transposases) were significantly more abundant in nasal cavity samples compared with fecal samples (Fig. S9; *p* < 0.05, Wilcoxon signed-rank test). qacEdelta and plasmids were most enriched in dust samples, whereas the insertion element IS91 was most abundant in the nasal cavity. Despite being the most abundant MGE type (75% of total MGEs), transposases exhibited comparable abundance across all sample types (Fig. 5a; *p* > 0.05), suggesting a conserved role in horizontal gene transfer regardless of the environmental niche.

Previous studies have shown that ARGs located within 5 kb of MGEs exhibit increased mobilization potential via MGE-mediated transfer [22]. In our study, 340 ARG‒MGE complexes—defined as contiguous genomic regions containing both ARGs and MGEs within 5 kb—were identified from contigs, encompassing 91 ARG subtypes and 34 MGE subtypes. Of these, 222 (65.29%) contained at least one potentially hazardous ARG (Tables S6, S7). The distribution of ARG‒MGE complexes varied across sample types, with 141 from nasal, 95 from fecal, and 104 from dust-derived contigs. Network analysis revealed that the transposase gene *tnpA* exhibited the highest degree of connectivity, linked to ARGs conferring resistance to aminoglycosides, MLS, tetracyclines, beta-lactams, and multidrug agents (Fig. 5b–d). Conjugative transposons of the Tn916 family consistently cooccurred with *tet*(M). Notably, the ARG‒MGE association patterns of the nasal and dust samples were similar, characterized by the frequent co-occurrence of *tnpA* with resistance genes against aminoglycosides (*AAC(3)-IV*, *APH(4)-Ia*, and *APH(3)-Ib*), tetracyclines (*tet*(Q), *tet*(S), and *tet*(Z)), MLS (*emtA*, *vga*(A), and *poxtA*), and beta-lactams (*mecI*, *mecR1*, and *mecA*). Plasmid-mediated ARGs were also more prevalent in nasal and dust samples, suggesting that these niches may harbor ARG profiles with greater mobility potential (Fig. 5b and d). In contrast, the enrichment of multidrug resistance genes such as *evgS*, *evgA*, *emrK*, and *emrY* was significantly greater in the fecal samples, reflecting the presence of niche-specific selection pressures (Fig. 5c). Collectively, these findings reveal distinct ARG–MGE association patterns across pig-associated niches.

### Genetic location of potentially hazardous ARGs

**Fig. 6:**
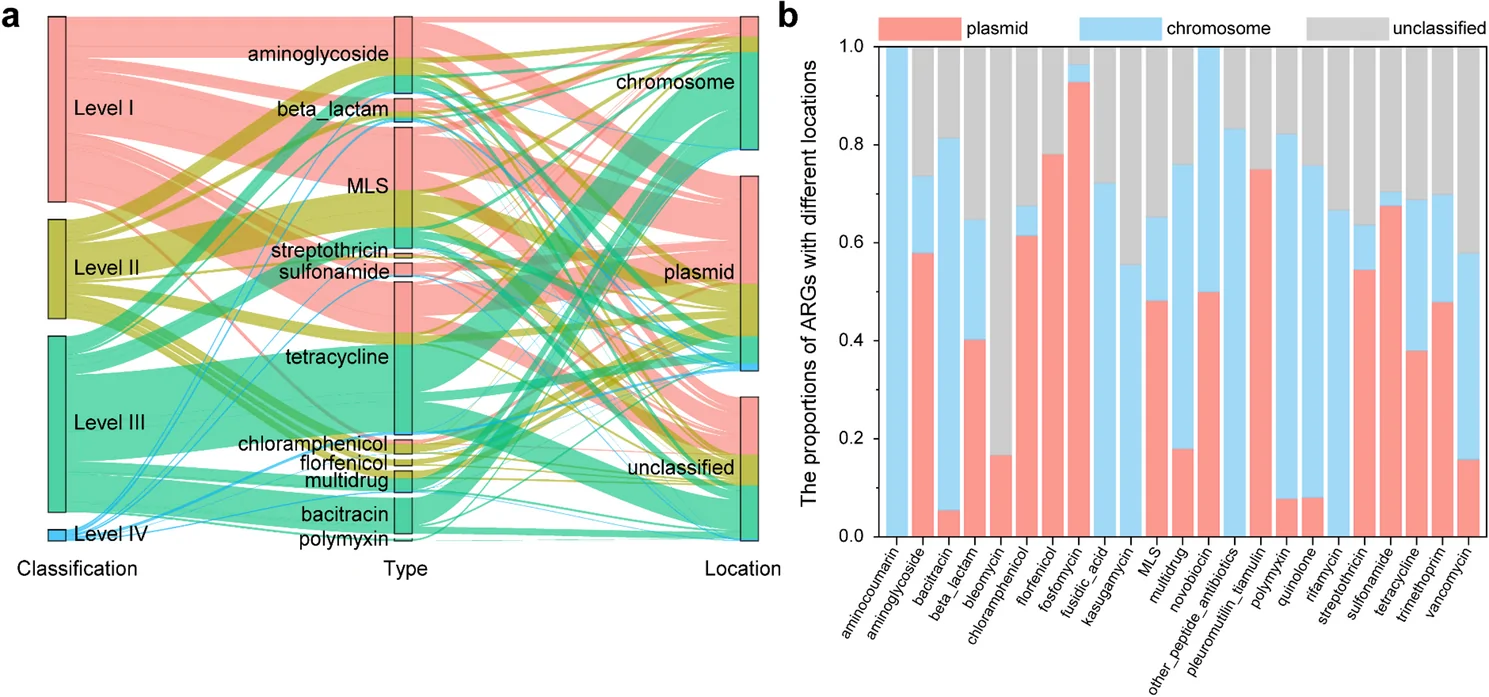
The genetic locations of ARGs. (**a**) Distribution of ARG types and genetic locations by risk level. The colors represent different risk levels. The lengths of the rectangles indicate the number of ARGs in the corresponding category. (**b**) The proportions of each ARG type on plasmids and chromosomes

To better understand the mobilization potential of ARGs, we next examined their genetic localization patterns. The distribution of potentially hazardous ARGs across risk levels revealed clear differences in terms of richness and resistance type (Fig. 6a). Among the 78 potentially hazardous ARGs, Level I and Level III ARGs were the most abundant, followed by Level II, whereas Level IV ARGs were relatively scarce. This pattern suggests that only a small subset of ARGs is host-specific (Level IV) and that a broader host range (Level III) does not necessarily imply higher mobility, underscoring the complexity of ARG distribution patterns and potential dissemination across niches. Distinct resistance patterns were observed across ARG risk categories. Level I ARGs were predominantly composed of aminoglycoside, MLS, and tetracycline resistance genes. In contrast, Level III ARGs consisted primarily of tetracycline resistance genes (49.5%), followed by bacitracin resistance genes. Among Level II ARGs, MLS resistance genes were most prevalent (37.57%), followed by aminoglycoside, tetracycline, and chloramphenicol resistance genes.

Substantial differences were also observed in the genomic localization patterns among the different ARG types (Fig. 6a and b). Genes conferring resistance to fosfomycin (92.86%), florfenicol (78.13%), pleuromutilin/tiamulin (75%), sulfonamide (67.61%), chloramphenicol (61.49%), aminoglycosides (58%), streptothricin (54.55%), MLS (48.23%), trimethoprim (47.95%), and beta-lactams (40.26%) were more commonly plasmid-borne. In contrast, genes conferring resistance to aminocoumarin (100%), bacitracin (75.96%), polymyxin (74.44%), quinolone (67.74%), rifamycin (66.67%), fusidic acid (72.22%), multidrug (57.93%), and vancomycin (42.11%) were primarily chromosomally encoded (Fig. 6b). Moreover, ARGs predicted to be plasmid-borne tended to be assigned to higher risk categories, with 55.31% classified as Level I and 27% as Level II, whereas the majority of chromosomal ARGs (80%) belonged to the lower-risk Level III or IV categories. This distribution pattern suggests that plasmid-associated ARGs may be more closely linked to clinically relevant resistance risks. Among the 340 identified ARG-MGE complexes, plasmid-borne ARG-MGE complexes (252/340, 74.12%) were predominant, significantly outnumbering those located on chromosomes (23/340, 6.76%). These findings suggest that plasmids may play a critical role in shaping the distribution and persistence of high-risk ARGs.

### Validation of resistome and mobilome patterns across distinct niches in an independent pig farm

**Fig. 7:**
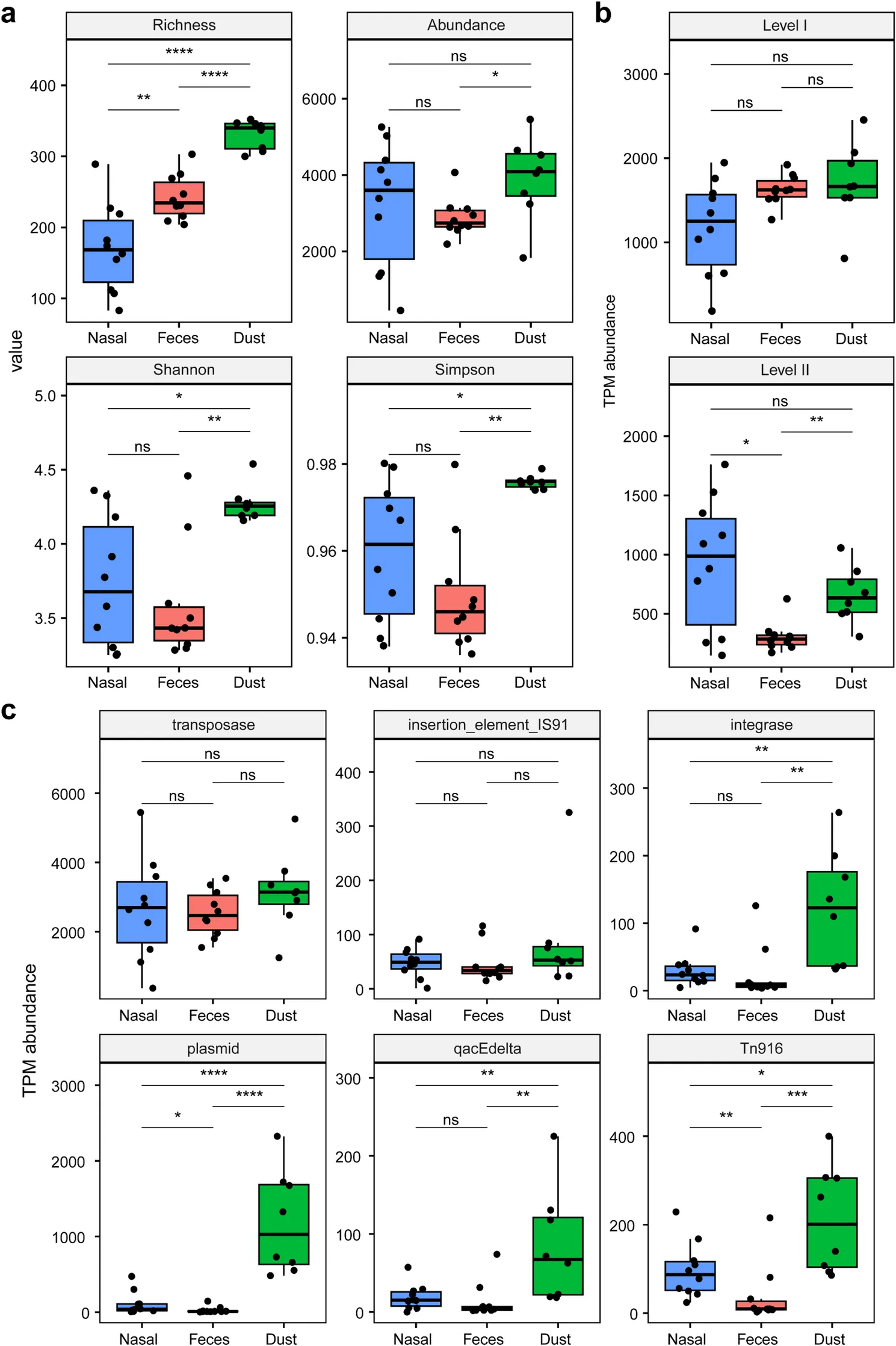
Comparison of resistome and mobilome patterns among the nasal cavity, feces and dust in a verification dataset. (**a**) Comparison of the richness (number), abundance, and α-diversity of ARG subtypes analysed in the verification dataset between different niches. *p* values were calculated using the Wilcoxon signed-rank test for comparisons between the nasal cavity and feces, and the Wilcoxon rank-sum test for comparisons involving dust. Asterisk indicates significance (*: *p* < 0.05; **: *p* < 0.01; ***: *p* < 0.001; ****: *p* < 0.0001). ns, no significant (*p* ≥ 0.05). (**b**) The abundance comparison of high-risk ARGs (Level I/II) across three niches. (**c**) Comparison of MGE types among the nasal cavity, feces and dust

To assess the robustness and generalizability of our findings, an independent validation dataset comprising 28 samples (including nasal and fecal samples from 10 pigs and associated dust samples) from a separate pig farm was analysed. Consistent with the discovery cohort, compared with the nasal and fecal samples, the dust samples presented the greatest ARG diversity, with significantly increased α diversity (as measured by the richness, Shannon, and Simpson indices) (Fig. 7a; Wilcoxon rank-sum test, *p* < 0.05). Although the number of ARGs (richness) was greater in the fecal samples than in the nasal samples, both the abundance and diversity (as indicated by the Shannon and Simpson indices) of the ARGs tended to be consistently greater in the nasal samples than in the fecal samples. However, the difference did not reach statistical significance (Fig. 7a; Wilcoxon signed-rank test, *p >* 0.05). Moreover, the abundance of Level II risk ARGs was significantly greater in both dust and nasal samples than in fecal samples (Fig. 7b). Furthermore, we observed that several key MGEs—including integrases, plasmids, qacEdelta, and the transposon Tn916—were also more enriched in nasal and dust samples, with a particularly notable accumulation in dust (Fig. 7c; *p* < 0.05). These results collectively confirm that dust and nasal cavities represent consistent and critical reservoirs of mobile ARGs in swine production environments.

## Discussion

### Pig nasal cavities and farm dust are critical reservoirs for ARGs

In this study, we conducted a comprehensive comparative analysis of the resistome and mobilome in pig nasal cavities, feces, and farm dust within the same commercial pig farm ecosystem using metagenomic analysis. Compared with feces, nasal and dust samples exhibited significantly greater ARG diversity and abundance. Most high-abundance MGE types, such as the insertion element IS91, integrase, plasmid, qacEdelta, and transposon Tn916, were also enriched in the nasal cavity and dust samples. These results suggest that the nasal cavity and farm dust of pigs are critical ARG reservoirs and deserve greater attention in the monitoring of AMR on pig farms. Current studies on the pig resistome focus primarily on the gut microbiota [47, 48], whereas studies on the respiratory tract and dust resistomes, especially those characterized by metagenomics, remain very limited.

We previously characterized the resistome in the lower respiratory tract of pigs using metagenomic approaches [11]. However, systematic studies on the resistome in the pig nasal cavity remain scarce. Notably, a study in yaks demonstrated that the nasal cavity harbors a high abundance and diversity of ARGs [53]. In pigs, Pirolo et al. [54] reported that the microbiota abundance is highest in the nostrils among all respiratory tract sites. These findings highlight the important role of the nasal microbiome as a critical ARGs reservoir. Another study demonstrated that compared with that of nonfarm workers, the nasal microbiota composition of pig farmers is more similar to that of pigs [55]. In our study, several ARG-enriched bacterial species were significantly more abundant in the nasal cavity than in the feces. In particular, *Glaesserella parasuis* (MAG623), a known porcine respiratory pathogen responsible for Glässer’s disease, was the most abundant species in the nasal cavity and harbored the greatest number of high-risk ARGs among all the MAGs analysed. These findings indicate that the nasal microbiome and the ARGs it carries may have the potential to spread to humans.

Airborne, dust-bound bacteria can spread across the farm and into the surrounding environment. In our study, we found that the α-diversity of ARGs was highest in dust samples, which also contained numerous ARGs that were abundant in nasal or fecal samples. Several additional studies have also demonstrated that dust harbors abundant ARGs, highlighting its significant role in the dissemination of ARGs [56, 57]. Luiken et al. [15] reported that ARG levels were higher in dust than in animal feces in a resistome study of European poultry and pig farms and that the resistome composition in farm dust was significantly correlated with that in animal feces. This finding suggests that part of the dust resistome originates from animal feces. Our results support this notion, as the fecal and dust samples had similar ARG compositions (Fig. 1c and d). However, we also found that the ARG-MGE co-occurrence patterns of dust samples were more similar to those of nasal samples than to those of fecal samples (Fig. 5). Collectively, these findings indicate that dust plays important roles as a vector and reservoir in the spread of ARGs, highlighting the role of the farm environment as a critical ARG reservoir.

It should be noted that dust samples were collected from distinct environmental locations within the same farm and therefore are not directly equivalent to pig-level biological replicates. Nevertheless, the enrichment of ARGs and MGEs in dust relative to feces was consistently observed across multiple dust-sampling locations and was further reproduced in an independent validation dataset from another pig farm, supporting the robustness of this niche-associated pattern.

### A risk assessment framework is essential for prioritizing high-risk ARGs from pig-related sources

ARG reservoirs from pig-associated niches may have direct implications for human health. Previous studies have shown that occupational exposure on farms can alter human skin and gut microbiota, as well as their associated resistomes, even following short-term exposure to livestock environments [58, 59]. Farm workers may serve as carriers of ARGs, potentially introducing these resistance genes into the broader community, thereby highlighting the long-term threat to public health posed by the misuse of antibiotics in agricultural settings. Therefore, assessing the health risks associated with pig-related ARGs is essential.

We identified 78 potentially hazardous ARGs and established a risk classification framework that categorizes these ARGs into four levels on the basis of three key criteria: host promiscuity, mobility, and human health risk. Compared with previously reported ARG assessment frameworks [21, 22], our framework is developed for pig-associated resistome, as it integrates prevalence, abundance, mobility, and pathogenicity potential based on pig-derived metagenomic data. Beyond several consensus high-risk ARGs identified across frameworks, such as *AAC(3)-IId*, *erm*(B), *tet*(L), and *poxtA*, our framework also identified several ARGs classified as high-risk (Level I/II) in this study but categorized as low-risk (Level III/IV) or overlooked in previous frameworks, such as *lnu*(C), *erm*(G), *ANT(3)-IIa*, and *tet*(Y). In particular, *lnu*(C), which is more specifically associated with lincosamide resistance although classified under the broader MLS category in the SARG database, was classified as Level III (Rank III) in Zhang Tong’s framework and Level IV (Q4) in Qian Haifeng’s framework. However, our results showed that it was detected in all the fecal samples, ranked among the top ten most abundant, and was highly abundant in the respiratory tract (Fig. 2e and f). In addition, *lnu*(C) was among the top twenty most abundant ARGs in dust. Moreover, *lnu*(C) displayed a broad host range, spanning bacterial species from multiple phyla, including *Clostridioides difficile*, a clinically important pathogen (Fig. 2e and Fig. S7). Li et al. [60] reported that *lnu*(C) is globally distributed among another pathogen, *Campylobacter coli*, on the basis of *C. coli* strains isolated from pigs, chickens, humans, and food across eight countries. Fang et al. [61] reported that exposure to microplastics and cypermethrin can lead to the enrichment of *lnu*(C) and promote the formation of a symbiotic network with other ARGs. Our data consistently revealed that the majority of *lnu*(C) genes (51.05%) were located on plasmids, whereas only 13.29% were located on chromosomes, supporting the transmissibility of *lnu*(C). These findings indicate that *lnu*(C) is a high-risk resistance gene in pig-associated environments and is becoming increasingly prevalent worldwide, highlighting the urgent need for its inclusion in future AMR surveillance and control strategies.

Notably, host assignment in the current framework was inferred from metagenomic contigs using Kraken 2, which may be uncertain for short contigs or highly similar genomic regions. Kraken 2 has been reported to show greater uncertainty at the species level, whereas classifications at the genus level or above are generally more reliable [40]. Because most potentially hazardous ARGs in our dataset were detected across multiple genera (66/78), the impact of this uncertainty on the overall host-promiscuity pattern and risk ranking is likely limited, although individual ARG–host links may still be affected. In addition, some pathogens are host-specific rather than zoonotic, while one of the indicators in our framework is based on human pathogenicity data from Qian Haifeng’s framework. This reliance limits the ability of the framework to fully capture the potential risk of ARGs to pigs. Future studies with expanded datasets and refined risk indicators are needed to address these limitations.

### ARG mobility is facilitated by plasmid carriage and MGE colocalization

Interestingly, we also identified ARGs located on the chromosome without any associated MGEs in the immediate flanking regions (within 5 kb upstream and downstream) but that displayed broad host distributions. These ARGs may not primarily rely on recent MGE-associated horizontal transfer. For example, *bacA* was detected in different species of both Bacillota and Pseudomonadota. Previous studies have suggested that *bacA* is an intrinsic gene in bacterial hosts and is widely distributed in various environments, such as pharmaceutical plant sites and rivers, whose abundance is often high, which further supports our observations [46, 62]. Another example is *tet*(37), which is frequently detected in various *Prevotella* species and in *Segatella copri* (formerly *Prevotella copri*) (Fig. 3c and Fig. S7), a core genus in the pig gut microbiota [63–65]. Hu et al. [66] reported that *tet*(37) was positively correlated with several *Prevotella* species, suggesting that it may confer a colonization advantage under antibiotic stress (e.g., tetracycline treatment). Overall, this classification framework provides a systematic characterization of ARGs distribution patterns and offers useful insights into their ecological distribution in farming systems and their potential public health risks.

The genetic localization of ARGs and their co-occurrence with MGEs are critical indicators for evaluating the mobilization potential of ARGs. Our results indicated that the majority of mobile ARGs (74.12%) were predicted to be located on plasmids and that compared with chromosomal ARGs, plasmid-borne ARGs were assigned higher risk levels under our assessment framework. Studies on resistomes in other environments have also suggested that MGE-associated ARGs are predominantly located on plasmids, whereas the abundance of chromosomally encoded ARGs is relatively low [62, 67]. Additionally, we found that plasmid-mediated ARGs were more prevalent in nasal and dust environments, with plasmids showing the greatest abundance in dust (Fig. 5). These findings underscore the essential role of plasmids in shaping ARG mobility patterns across different environments and emphasize that dust may represent an environmental reservoir enriched in plasmid-associated ARGs.

## Conclusions

In summary, this study identified pig nasal cavities and dust as critical yet overlooked AMR reservoirs and revealed significantly greater ARG diversity, abundance, and mobility in these areas than in feces. We further developed an enhanced ARG risk assessment framework that incorporates host promiscuity, mobility, and human health risks to better prioritize risk in swine-associated resistomes. Notably, compared with chromosomal ARGs, plasmid-borne ARGs, which were more abundant in nasal and dust samples, were inferred to have higher potential health risks under our assessment framework. These findings provide important reference data for monitoring resistome risk in pig production, although further large-scale studies are needed to improve generalizability.

## Supplementary Information

Below is the link to the electronic supplementary material.

Supplementary Material 1

Supplementary Material 2

## Data Availability

The metagenomic sequencing data are publicly available in the Genome Sequence Archive (GSA) database with BioProject number PRJCA041611 (direct access link: https://ngdc.cncb.ac.cn/gsa/browse/CRA027749).
