# Supplementary material for: Metagenomic analysis reveals resistome characteristics and high-risk resistance genes in the pig nasal cavities, feces, and farm dust
Source: Anim Microbiome. 2026 Jun 12;8:94. doi: 10.1186/s42523-026-00589-y (PMC13487949; doi:10.1186/s42523-026-00589-y)
Supplement: Supplementary file 1 [file 42523_2026_589_MOESM1_ESM.docx]

**Supplementary information for**

**Metagenomic analysis reveals resistome characteristics and high-risk resistance genes in the pig nasal cavities, feces, and farm dust**

Yangfan Yu^1,#^, Hai Wu^1,#^, Honghu Ji^2#^, Yuhan Hu^1^, Yihuan Fang^1^, Yuntian Lin^1^, Yong Zhang^1^, Yunyan Zhou^1*^

^1^ *College of Pharmaceutical Science & Collaborative Innovation Center of Yangtze River Delta Region Green Pharmaceuticals, Zhejiang University of Technology, Hangzhou 310014, China*

^2^ *Jinhua Academy of Agricultural Sciences，Jinhua 321017, China*

^#^ These authors contributed equally to this work.

^*^Corresponding author:

Yunyan Zhou (E-mail: zyy@zjut.edu.cn)

Submitted to **Animal Microbiome**

**The supporting information contains 9 figures and 8 tables.**

**Supplementary Figures**

**Fig. S1. The abundance of different types of antibiotic resistance genes (ARGs) in each of 36 samples.** The figure displays only the top 10 most abundant ARG types, with all remaining low-abundance types grouped into an "others" category.

**Fig. S2. The risk assessment of ARGs in the nasal cavity, feces, and dust.** **a.** The distributions of genes of different risk ranks in each resistance type. **b.** The distribution of shared and specific ARGs among the three niches in each risk rank. The red numbers represent the total count of genes. **c.** The abundance of ARGs of different risk ranks in the nasal, feces, and dust niches.

**Fig. S3.** **Distribution of potentially hazardous ARGs in different niches. a.** The distribution of Rank I ARG subtypes (n=30) within each niche. **b.** The distribution of Rank II ARG subtypes (n=26) within each niche. **c.** The distribution of high-abundance ARG subtypes (n=44), which were screened using a criterion of the top 20 for average abundance in each niche.

Fig. S4. The cumulative abundance ratio of risk ARG candidate sets in nasal cavity, feces and dust. "Top 20" represents the union of the top 20 most abundant ARGs in each group. "Top20_union" represents the union of the top 20 most abundant ARGs across groups. “Rank” denotes the final set of 78 78 potentially hazardous ARGs included in the risk assessment framework.

Fig. S5. Comparison of the classification of the 78 potentially hazardous ARGs identified in this study with Qian Haifeng’s and Zhang Tong’s frameworks. Risk levels in this study are denoted as Level I–IV, whereas those in Qian Haifeng’s framework and Zhang Tong’s framework are denoted as Q1–Q4 and Rank I–IV, respectively.

**Fig. S6.** **The distribution of ARGs in host bacteria at different taxonomic levels.** Different colors represent different taxonomic levels, and the height of the rectangles represents the number of ARGs. Only taxa harboring more than twenty ARGs were shown.

**Fig. S7.** **The distribution of potentially hazardous ARGs in MAGs.** The red diamonds represent the ARGs; Circles represent MAGs, with different colors indicating different bacterial genera. The connections between MAGs and ARGs represent the number of ARG copies carried by each MAG. Black lines indicate that the MAG carries one copy of the corresponding ARG, while pink lines indicate two copies.

**Fig. S8. The proportion of different Mobile genetic elements (MGEs) types.** Six main types were particularly displayed.

**Fig. S9.** **Comparison of MGE types between the nasal cavity and feces.** The Wilcoxon rank-sum test was used for comparison between the nasal cavity and feces. *p* < 0.05 was set as the significance threshold. The Wilcoxon signed-rank test was used for pairwise comparisons. Asterisk indicates significance (*: *p* < 0.05; **: *p* < 0.01; ***: *p* < 0.001; ****: *p* < 0.0001). ns, no significant (*p* ≥ 0.05). Boxplots show the median, 25th, and 75th percentiles. The lower and upper boundaries of whiskers indicate the minima and maxima, respectively. Each dot represents a sample.

**Supplementary Tables**

Supplementary Tables are provided by an Excel document.

**Table S1. The annotation and classification of ARGs**

**Table S2. The number of ARGs carried by species**

**Table S3. The number of ARG subtypes carried by MAGs**

**Table S4. The distribution of potentially hazardous ARGs in MAGs**

**Table S5. The annotation and classification of ARGs**

**Table S6. The information of ARG and MGE on the contig where ARG and MGE coexist**

**Table S7. The genetic localization of 340 ARG-MGE complexes**

**Table S8. Detailed information on 78 potentially hazardous ARGs for assessing risk levels**
